# Dr. George Regnoldi

**Published:** 1860-01

**Authors:** 


DR. GEORGE REGNOLDI, Professor of Surgery in the University of Florence, died recently, after having acquired a world-wide reputation as a teacher and a skillful operator.

